# Differential Effects of First and Third Trimester HbA1c Levels on Neonatal Weight and Placental Mediation in Type 1 Diabetes

**DOI:** 10.3389/fendo.2025.1595584

**Published:** 2025-06-16

**Authors:** Mohammed Bashir, Adeel Khan, Fateen Ata, Manar E. Abdel-Rahman, Faten Eltaher, Gernot Desoye, Justin C. Konje, Abdul-Badi Abou-Samra

**Affiliations:** ^1^ Qatar Metabolic Institute, Hamad Medical Corporation, Doha, Qatar; ^2^ National Diabetes Center, Women Wellness and Research Centre, Hamad Medical Corporation, Doha, Qatar; ^3^ Department of Internal Medicine, Cleveland Clinic Akron, Akron, OH, United States; ^4^ Endocrine Department, Hamad Medical Corporation, Doha, Qatar; ^5^ Department of Public Health, College of Health Sciences, Qatar University, Doha, Qatar; ^6^ Department of Obstetrics and Gynaecology, Women Wellness and Research Centre, Hamad Medical Corporation, Doha, Qatar; ^7^ Department of Obstetrics and Gynaecology, Medical University of Graz, Graz, Austria; ^8^ Department of Obstetrics and Gynecology, Feto Maternal Centre, Doha, Qatar; ^9^ Department of Obstetrics and Gynecology, Weill Cornell Medicine, Doha, Qatar; ^10^ Department of Obstetrics and Gynaecology, University of Leicester, Lecister, United Kingdom

**Keywords:** type 1 diabetes mellitus, pregnancy, placenta, meditation, neonatal weight

## Abstract

**Context:**

In women with type 1 diabetes (T1D), the effects of glycaemic control on neonatal weight and the role of the placenta are not fully understood.

**Objective:**

This study explores the relationship between glycaemic control, neonatal weight, and placental weight.

**Design:**

A retrospective observational longitudinal study of pregnant women with T1DM.

**Setting:**

The study included 265 women with T1D. The target for the first A1c was set at ≤ 7.0%, while the target for the last A1c was ≤ 6.5%. The cohort was divided into four groups based on whether they achieved their target A1c (T) or had levels higher than the target (H) at each end. These groups were classified as Target-Target (T-T), Target-High (T-H), High-Target (H-T), and High-High (H-H). For the secondary objective, we included 154 women for whom placental weight data were available.

**Main outcome:**

We assessed the association between firstA1c, lastA1c, and neonatal weight, examining the mediation effect of the placenta.

**Results:**

The mean age of the participants was 29.4 years (SD 4.6), and the mean T1DM duration was 14.1 years (SD 7.1). The median neonatal weight was highest in the T-H group (3.56 kg) and lowest in the H-T group (3.20 kg) (p=0.009). FirstA1c was negatively correlated with neonatal weight (β-coefficient -150.9, p < 0.01), whereas lastA1c positively correlated (β-coefficient 162.5, p < 0.01). The association with firstA1c disappeared when correcting for placental weight, while lastA1c remained significant. The placenta mediates 65% of the impact of firstA1c on neonatal weight

**Conclusions:**

Poor glycaemic control early in pregnancy is linked to lower neonatal birth weight, while poor control in the third trimester is associated with higher birth weight. These findings emphasize the importance of maintaining adequate glycaemic control before and during early pregnancy for better health outcomes.

## Introduction

The incidence and prevalence of type 1 diabetes mellitus (T1DM) are increasing worldwide (1, 2). There were 8.4 million people with T1DM in 2021, and the International Diabetes Federation (IDF) projects this to rise to 17 million in 2040 (2). With a peak incidence at 10–14 years, more women of reproductive age are expected to have T1DM. The prevalence of T1DM in pregnancy is, therefore, expected to increase. Indeed, the incidence of pre-pregnancy T1DM has doubled between 1990-2020 (3). In Qatar, 0.2% of all pregnancies are affected by T1DM (4). Pregnancies with T1DM are associated with an increased risk of maternal and fetal complications such as congenital malformations, pre-term labor, pre-eclampsia, Cesarean section ( C-sections), large for gestational age (LGA), small for gestational age (SGA), and intra-uterine fetal death (IUFD) (5–7). Apart from glycaemic control, pre-gravid obesity, excessive gestational weight gain, and smoking are factors associated with poor pregnancy outcomes (8).

While improvements in pre-pregnancy care have resulted in a lower incidence of congenital malformation in women with DM, there has been a paradoxical increase in the rate of LGA (9). Glycaemic control plays a central role in determining birth weight. Most studies have shown that elevated third-trimester glycated hemoglobin (HBA1c) was associated with increased fetal weight (5, 10–14). However, there are contradicting data on the association between first-trimester HBA1c and birth weight, with some studies showing increased birth weight with higher pre-pregnancy HBA1c and others showing a reduced fetal weight with higher pre-pregnancy HBA1c (10, 15, 16).

**Figure 1:**
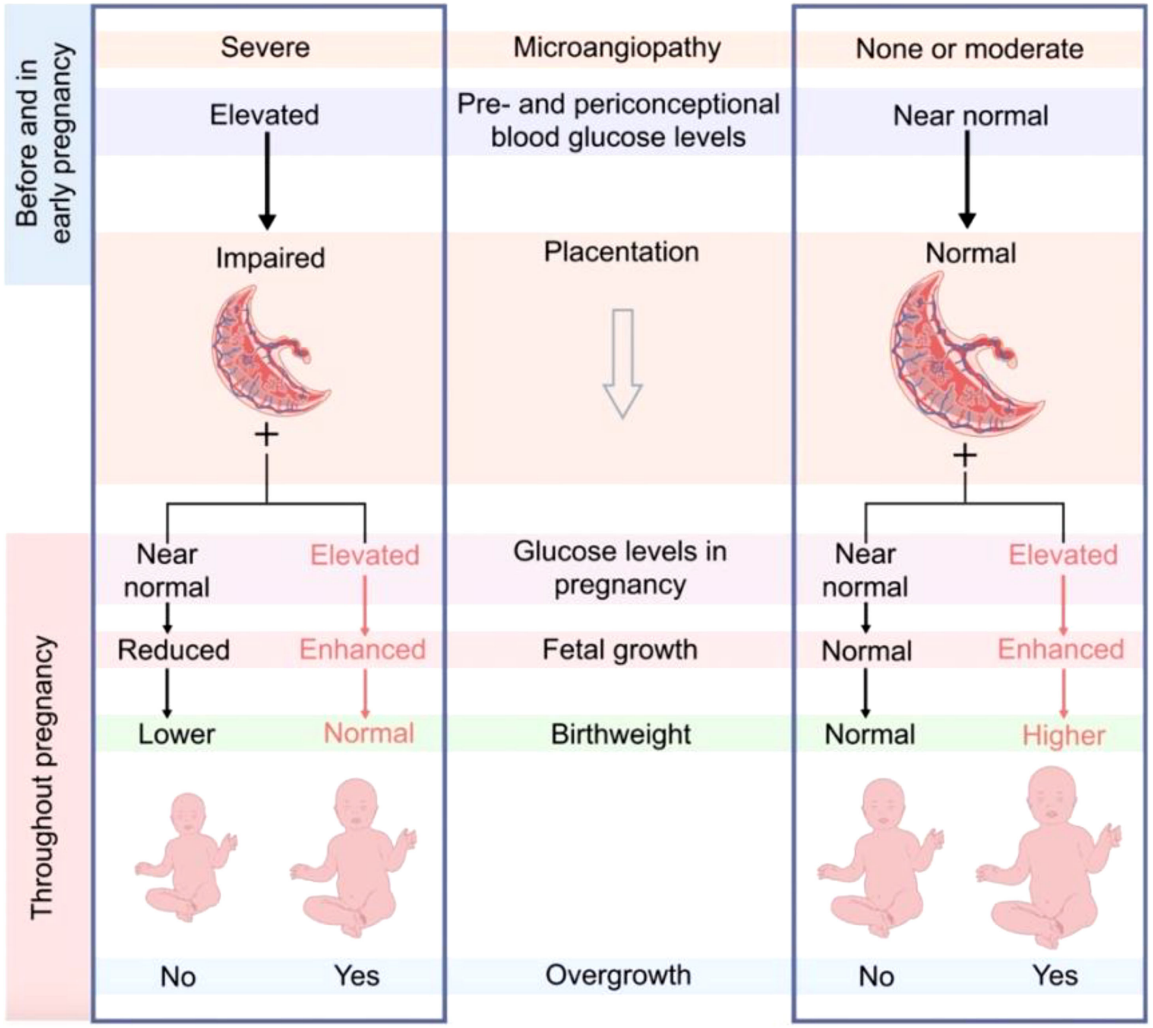
Desoye et al. Proposal on the interaction of early and late glycaemic control and placentation. http://creativecommons.org/licenses/by/4.0.

The placenta plays a central role in the rates of nutrient transfer to the fetus. Desoye et al. suggested that pre-pregnancy glycaemic control is central to placental development (9). They hypothesized that poor glycaemic control in early pregnancy is associated with poor placentation, while adequate control is associated with healthy placentation (**Figure 1**). Hence, in those with adequate glycaemic control, mild hyperglycemia during pregnancy may be associated with increased birth weight, while in those with poor pre-pregnancy glycaemic control, adequate glycaemic control during pregnancy might be associated with reduced birth weight (9). Data from a randomized control trial (RCT) showed that birth weight is higher in those with good placental health and sub-optimal third-trimester glycaemic control (17).

This proof of concept study aims to investigate the intricate relationship between glycaemic control, fetal weight, and placental weight during pregnancy. The primary objective is to analyze the impact of HbA1c levels recorded during early pregnancy and the third trimester on fetal birth weight. By examining these critical time points, the study aims to determine how varying glycaemic control influences the growth and development of the fetus. The second objective is to assess the role of the placenta in mediating the effects of glycaemic control on fetal body weight and the correlation between placental weight, maternal glycaemic control and fetal birth weight. This involves evaluating the correlation between placental weight, maternal glycemic control, and fetal birth weight. Through this detailed examination, the research hopes to identify potential implications for pre-conception and prenatal care for managing women with T1DM.

## Methods

### Study design and population

This was a retrospective observational longitudinal study of pregnant women with T1DM who attended the National Diabetes Centre in the Women Wellness and Research Centre (WWRC) at Hamad Medical Corporation (HMC). The WWRC is the largest maternity hospital in the country and manages between 16000 and 18000 deliveries per year. In turn, the NDC-WWRC is the leading tertiary center that provides care for pregnant women with diabetes. All women with T1DM are managed according to the Qatar national guidelines (18). Most women with T1DM are treated with basal-bolus insulin regimen while very few use continuous insulin infusion. In women on continuous glucose monitoring (CGM), we aim for 70% of the readings within the time in range (defined as glucose between 3.5-7.8 mmol/l ). In women using capillary blood glucose, we aim for fasting glucose of ≤ 5.3 mmol/l and 2 hours post meals of ≤ 6.7 mmol/l.

The primary objective is to analyze the impact of HbA1c levels recorded during early pregnancy and the third trimester on fetal birth weight. The second objective is to assess the role of the placenta in mediating the effects of glycaemic control on fetal body weight. To test the hypothesis that the first and third-trimester HBA1C levels influence birth weight, we studied 265 women with T1DM who delivered at the WWRC between 2014-2021 (19). We excluded women who booked for antenatal care (ANC) after the 20^th^ week of gestation or had a miscarriage. We have previously reported the pregnancy outcomes in this cohort in a separate paper (19). To investigate the role of the placenta on the interaction between the first and last trimester HBA1c levels and birth weight, we studied 154 women in whom placental weight was available. The study was approved by the Institutional Review Board, Medical Research Centre of Hamad Medical Corporation (MRC 01-21-1035). Due to the study's retrospective nature, the informed consent form was waived by the Institutional Review Board. The study was carried out in line with the Helsinki Declaration.

### Data collection and definitions

All data were extracted from the electronic medical records (Cerner), which all public health care providers in Qatar use. For this study, we obtained the following information at baseline: age at conception, nationality, pre-gravid weight, pre-gravid height, age of onset of T1DM, HbA1c level at conception, third trimester HBA1c level, birth weight, and placental weight.

The patient's ethnicity was classified into Qatari, non-Qatari Arab (residents of the Middle East and North Africa Region), and others. Pre-pregnancy weight is recorded during the first visit based on the patient's self-report. We used the last height recorded before conception or the height recorded in the first antenatal visit to calculate the body mass index (BMI). We categorized the women as normal weight, overweight, and obese based on ethnic-specific BMI cut-off points (20).

Glycaemic targets were defined as HBA1c ≤ 7.0% in the first trimester (0–13 weeks) and ≤ 6.5% in the third trimester (28–40 weeks) based on the Qatar national guidelines (18). For the first HBA1c, we included the HBA1c measured within 3 months prior to conception or 8 weeks after conception. For the third trimester HBA1c, we included the last HBA1c measured after 32 weeks’ gestation. We categorized BMI as normal (<25kg/m^2^), overweight (BMI 25kg/m^2^ -29.9/m^2^) and obese ( BMI ≥30kg/m^2^). The women were categorized based on glycaemic control in the first trimester and at delivery into four groups; (a)Target-Target (T-T) (those who achieved targets at booking of ≤ 7.0% and at delivery ≤ 6.5%) (b)Target-High (T-H) (those with target levels at booking ≤ 7.0% but at delivery it was >6.5% ) (c) High-Target ( H-T) (those HBA1c >7.0% at booking but ≤ 6.5%) at delivery ) and (d) High-High ( H-H) (those who did HBA1c >7.0% at booking and > 6.5% at delivery). For the cohort with placental data, we examined the potential mediation effect of the placental weight on the neonatal birth weight.

We defined macrosomia was defined as a birth weight greater than 4000 grams, LGA as fetal weight more than the 90^th^ percentile for the gestational age, and SGA as fetal weight less than the 10^th^ percentile for the gestational age. We calculated gestational weight gain based on the reported pre-pregnancy weight and the weight recorded at delivery. We defined preterm labor was defined as delivery before the 37th week of gestation.

### Statistical analysis

**Figure 2:**
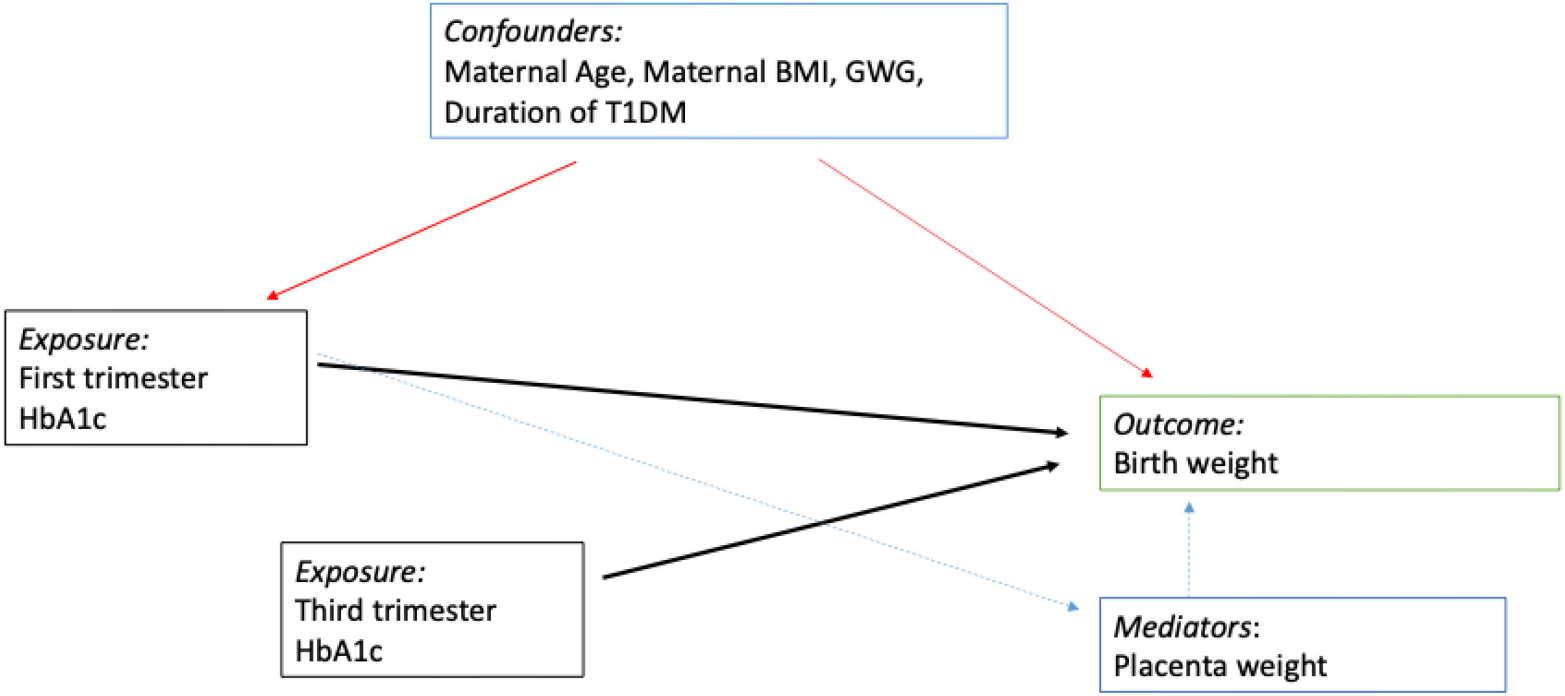
Directed Acyclic Graph. We propose that the placental weight is on the causal pathway between the first trimester A1c and the neonatal birth weight. However, the third trimester A1c has a direct effect on neonatal birth weight. Red arrows for confounding factors, black arrows are for direct causal effect and green for a mediator effect.

We used STATA 15 for statistical analysis. Continuous data are summarized as mean (SD) and median (IQR) as appropriate, while discrete data are summarized as percentages. Continuous variables were checked for normal distribution using the Skewness and Kurtosis test. Kruskal-Wallis test was used to compare the neonatal birth weight across the four glycaemic groups (21). A *post-hoc* analysis for neonatal birth weight (grams) was performed using Dunne's test (22). To investigate the causal relationship between first-trimester HBA1c (firsta1c) and third-trimester HBA1c (lasta1c), a Directed Acyclic Graph DAG was used (**Figure 2**). As seen from the DAG, we proposed that the placenta is on the causal pathway between the firsta1c, and neonatal birth weight. We performed four multivariable linear regression models to assess the association between *neonatal birth weight (as the dependent variable)* and *firsta1c* and *lasta1c (independent variables)*. Model 1 was a crude model that included firsta1c and lasta1c. Model 2 was adjusted for age and nationality. Model 3 was further adjusted for BMI, duration of diabetes, gestational weight gain (GWG), and gestational age at delivery (GA). Model 4 included an additional adjustment for the logarithmic transformation of placenta weight. This transformation was done to satisfy the normality assumption of the model.

Structural equation models were constructed to assess mediation and examine the direct and indirect effects (via *placental weight in grams*) of *firsta1c* and *lasta1c* on fetal *weight in grams*. Regression diagnostics were performed to assess the linearity assumption using residual versus predictor plots, normality assumption using Q-Q plots, constant variance assumptions using residual versus fitted values plots, and White's test. The models were assessed for specification error and multicollinearity using the variance inflation factor. Influential observations were assessed using the dfbeta statistics. Sensitivity analysis was performed to evaluate the impact of potential influential observations on regression results. We considered p<0.05 to be statistically significant.

## Results

We included 265 women with T1DM; placental weight data was available for 154 women. As shown in **Table 1**, the mean age (SD) was 29.4 (4.6) years, the mean (SD) duration was 14.1 (7.1) years, the mean BMI (SD) was 27.2 (5.2) kg/m^2^), and 62.5% of women were either overweight or obese. The mean (SD) HBA1c dropped from 7.9 (1.4) % in the first trimester to 6.9 (1.1) % in the third trimester. Similarly, the proportion of women within glycaemic targets improved from 28.8% in the first trimester to 40.0% in the third trimester. As shown in **Table 1**, 20% were at target in both the first and third trimesters (T-T), 8.7% were at target in the first trimester but not in the third trimester (T-H), 20% were above target in the first trimester but at target in the third trimester (H-T), and 51.3% were above target at first and third trimesters (H-H). **Supplementary Table 1** shows the demographics and pregnancy outcomes of the four groups (23).

**Table 1:** Baseline characteristics of the 265 women with T1DM included in the study.

Variable	Results (265 women)	
Age, mean (SD)	29.4 (4.6) years	
Ethnicity, n (%)	Qatari	133 (50.2%)
	Arab	115 (43.4%)
	Others	17 (6.4%)
Duration of DM, mean (SD)	14.1 (7.1) years	
Weight, mean (SD)	69.6 (13.8) kg	
BMI, mean (SD)	27.2 (5.2) kg/m^2^	
Weight Category, n (%)	Normal	97 (37.4%)
	Overweight	85 (32.8%)
	Obese	77 (29.7%)
1^st^ Trimester A1C, mean (SD)	7.9 (1.4) %	
3^rd^ Trimester A1c, mean (SD)	6.9 (1.1) %	
Total daily dose of insulin/kg, mean (SD)	0.97 (0.43) units/kg	
GWG/week, mean (SD)	290 (188) grams/week	
Excessive GWG, n (%)	165/259 (63.7%)	
1^st^ Trimester A1c targets (≤ 7.0%), n (%)	76 (28.8%)	
3^rd^ Trimester A1c targets (≤ 6.5%), n (%)	106 (40%)	
T-T, n (%)	53 (20.0%)	
T-H, n (%)	23 (8.7%)	
H-T, n (%)	53 (20.0%)	
H-H, n (%)	136 (51.3%)	
*GWG (Gestational weight gain)*		
*T-T (Target- Target)*		
*T-H (Target-High)*		
*H-T (High-Target)*		
*H-H (High-High)*		

**Figure 3:**
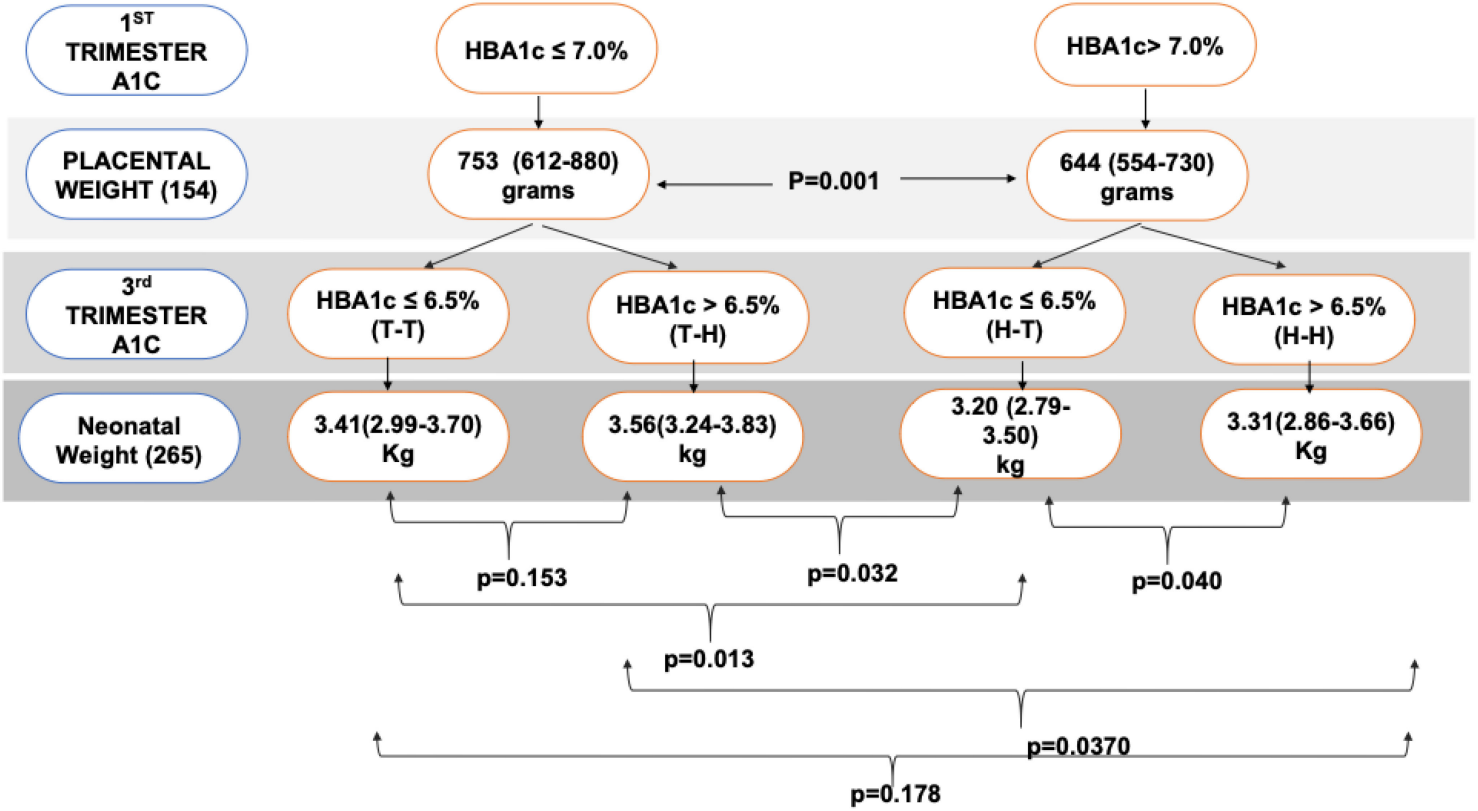
Shows the median (IQR) of the neonatal weight across the four glycaemic groups.

As shown in **Figure 3**, the median (IQR) of fetal weight was higher in the T-H group [3.56 kg(3.24-3.83)], followed by the T-T group [ 3.41 kg(2.99-3.70)]; then H-H[ 3.31 kg(2.86-3.66)] and T-H had the lowest birth weight [ 3.20 kg( 2.79-3.50)]; p=0.009. *Post-hoc* analysis showed that the H-T group has significantly lower birth weight when compared with T-T, T-H, and H-H, p= 0.013, 0.032, and 0.040, respectively.

**Table 2** shows the demographic of the study population with placental data. **Table 3** presents the results of the regression analysis. Model 1 showed that firsta1c was negatively associated with neonatal weight (β-coefficient -150.9 [ 95% CI -244.6, -57.2], p<0.01) while thirda1c was positively associated with neonatal weight (β-coefficient 162.5 [ 95% CI 44.8,280.2], p<0.01). This relationship remained unchanged in Models 2 and 3. However, after correction for log (placenta) in Model 4, the relationship between the firsta1c and neonatal weight was lost, while that with thirda1c remained significantly positive. This suggests that the placenta is the main mediator of the negative effect of the firsta1c on the neonatal weight.

**Table 2:** Baseline characteristics of the 154 women with T1DM with data on placental weight.

Variable	Results (154)	
Age (years), mean (SD)	30.1 (4.4)	
Ethnicity	Qatari	84 (54.5%
	Arab	58 (37.7%)
	Others	12 (7.8%)
Infant weight (grams), mean (SD)	3151.7 (668.8)	
First A1C (%), mean (SD)	8.0 (1.4)	
Last A1C (%), mean (SD)	7.0 (1.1)	
BMI Kg/m2, mean (SD)	28.2 (5.6)	
Duration (years), mean (SD)	15.5 (7.1)	
Gestational weight gain (Kg), mean (SD)	10.4 (6.7)	
Gestational age (weeks), mean (SD)	36.4 (2.1)	
Placental weight (grams), mean (SD)	675.1 (167.4)	
Logarithm of Placental weight (grams), mean (SD)	6.5 (0.2)	

**Table 3:** Linear regression analysis of infant weight in grams as a function of First A1C, Last A1c and other covariates.

Model 1			
	β−Coefficient	95% CI	P-Value
Frist A1c (%)	-150.9	-244.6, -57.2	**p<0.01**
Last A1c (%)	162.5	44.8,280.2	**p<0.01**
***Adjusted R -Square***	***0.06***		
Model 2			
	β−Coefficient	95% CI	P-Value
Frist A1c (%)	-152.1	-244.0, -60.3	**<0.01**
Last A1c (%)	164.2	47.9,280.5	**<0.01**
Maternal age (Years)	-22.5	-46.1,1.1	0.06
Qatari Vs Non-Qatari	-253.9	-461.1, -46.6	**<0.05**
***Adjusted R -Square***	***0.1***		
Model 3			
	β−Coefficient	95% CI	P-Value
Frist A1c (%)	-132.6	-217.4, -47.7	**<0.01**
Last A1c (%)	219	111.6,326.4	**<0.001**
Maternal age (Years)	-4.8	-28.5,19.0	0.74
Qatari Vs Non-Qatari	-241.4	-424.5, -58.3	**<0.05**
BMI (Kg/m2)	10.6	-8.4,29.5	0.281
Duration (Years)	3.5	-10.6,17.5	0.756
Gestational weight gain (Kg)	12.6	-2.1,27.3	0.081
Gestational age (Years)	176.9	129.3,224.4	**<0.001**
***Adjusted R -Square***	***0.39***		
Model 4			
	β−Coefficient	95% CI	P-Value
Frist A1c (%)	-47.2	-121.0,26.7	0.185
Last A1c (%)	166.9	76.7,257.2	**<0.001**
Maternal age (Years)	-3.5	23.3,16.2	0.794
Qatari Vs Non-Qatari	-156.5	-310.1,-2.8	**<0.05**
BMI (Kg/m2)	19.0*	3.1,34.9	**<0.05**
Duration (Years)	2.4	-9.2,14.1	0.766
Gestational weight gain (Kg)	1.7	-10.8,14.2	0.868
Gestational age (Years)	131.8	90.6,173.0	**<0.001**
Log Placental Weight (grams)	1345.9	998.0,1693.8	**<0.001**
***Adjusted R -Square***	***0.58***		

Bold indicates statistically significant.

Mediation analysis (**Supplementary Tables 2**-**4**) shows that 64% of the effect of the firsta1c on neonatal weight was mediated through the placenta (23).

## Discussion

Our study supports the complex interaction between pre-pregnancy glycaemic control and birth weight, as proposed by Desoye et al. We have shown that neonatal birth weight varies based on the level of glycaemic control in the first and third trimesters. Specifically, we observed the lowest fetal weight in individuals with poor early glycaemic control but achieved glycaemic targets in the third trimester (H-T group). Our findings suggest that poor early glycaemic control is linked to lower birth weight, primarily due to inadequate placentation. On the other hand, elevated third-trimester HbA1c levels are independently associated with increased birth weight.

Our findings support the results of other studies. An *in-vitro* study compared the placental tissues of 12 women with T1DM with 11 controls showed a decrease in the protein levels of the proliferation markers, cell cycle regulators, and cell invasion cycle in those with T1DM (24). Furthermore, under hyperglycemic conditions, the number of trophoblast cells was reduced (24). A longitudinal analysis of 157 women with T1DM enrolled in the CONCEPTT trial assessed the placental function at baseline and again at 24- and 34-weeks' gestation by measuring placental growth factor (PlGF) and soluble fms-like tyrosine kinase 1 (sFlt-1) (17). The study reported improved placental health with adequate pre-conception glycaemic control and reduced placental health with inadequate pre-conception glycaemic control (17). Infants of women with suboptimal glycemia were heavier when the placenta was healthy and lighter when the placenta was unhealthy (17).

Adequate placentation is critical for a healthy pregnancy. People with T1DM are known to be at higher risk of microangiopathy, resulting in retinopathy, nephropathy, and peripheral neuropathy. The cumulative exposure to hyperglycemia mainly determines the risk and extent of microangiopathy. In a large cohort study of people with T1DM, hyperglycemia, duration of diabetes, and hypertension were the main risk factors for microangiopathy (25). Poor placentation is likely another manifestation of microangiopathy in women with T1DM (9). Another possible factor of poor placentation is hypertriglyceridemia, an independent risk of microangiopathy in people with T1DM (26). Pre-conception hypertriglyceridemia secondary to poor glycaemic control and obesity is a known association with poor placentation and a higher risk of pre-eclampsia (27).

Birth weight is associated with short and long-term metabolic complications. A large cohort study of women with T1DM showed that the risk of IUFD is increased by 3–6 folds with either LGA or SGA infants (8, 28). Regardless of the birth weight, offspring of mothers with T1DM have lower muscle mass and higher fat mass, which increases the future risk of metabolic disorders (29). SGA infants have normal visceral fat but low total and subcutaneous fat (30). Accelerated weight gain in the first two years leads to more visceral fat deposition in SGA infants, which increases their future risk of hypertension and T2DM (30). On the other hand, LGA offspring are born with increased total body fat and are at higher risk of childhood obesity, T2DM, and cardiovascular disease (31). Furthermore, Infants in the H-H group are more likely to have intrauterine hypoxia due to overnutrition with impaired placental function. A small study showed that the offspring of women with T1DM who had intra-uterine hypoxia have higher measures of adiposity and glycemia in adulthood than those with no hypoxia (32). While those with H-T are at higher risk of SGA, it is not clear if they exhibit the same body composition of increased visceral and reduced subcutaneous fat. Hence, thriving to achieve normal placentation and maintain a normal fetal weight is critical to reducing the short- and long-term complications for offspring of mothers with T1DM.

Our findings have significant clinical implications. It is clear that adequate glycaemic management during pregnancy is insufficient to achieve favorable pregnancy outcomes. Hence, pre-conception care should be offered to all women with T1DM at reproductive age. Furthermore, adequate glycaemic control should be achieved and maintained throughout pregnancy. For those women who conceive with suboptimal glycaemic control, third-trimester glycaemic targets should be adjusted according to the fetal size to avoid restricted fetal growth.

The main limitation of the study is its retrospective nature. We lacked data on critical confounding factors such as parity, smoking, history of hypertension, history of microvascular complications, and albuminuria. Additionally, we did not have trimester-specific gestational weight gain data. However, the study's strength lies in the relatively large number of subjects and the careful analysis.

In conclusion, our findings showed that poor early glycaemic control is linked to lower neonatal birth weight, while poor third-trimester control is associated with increased fetal birth weight. The impact of early glycaemic control on neonatal weight is mainly mediated through poor placentation. These findings emphasize the crucial role of pre-conception and early glycaemic control in pregnancy outcomes.

## Data Availability

The raw data supporting the conclusions of this article will be made available by the authors, without undue reservation.
