# Supplementary material for: Differential Effects of First and Third Trimester HbA1c Levels on Neonatal Weight and Placental Mediation in Type 1 Diabetes
Source: Front Endocrinol (Lausanne). 2025 Jun 16;16:1595584. doi: 10.3389/fendo.2025.1595584 (PMC12206601; doi:10.3389/fendo.2025.1595584)
Supplement: Supplementary file 1 [file Table1.docx]

Supplementary Table 1:- Baseline characteristics and pregnancy outcomes of the four glycaemic groups.

|  | **T-T (53)** | **T-H (23)** | **H-T (53)** | **H-H (136)** | **P value** |
| --- | --- | --- | --- | --- | --- |
| **Age (years), mean (SD)** | 29.4 (4.2) | 27.8 (3.6) | 30.8 (5.4) | 29.1 (4.6) | 0.0448 |
| **Ethnicity , n(%)** | Qatari (52.8%) | Qatari (34.8%) | Qatari (43.4%) | Qatari (54.4%) | 0.062 |
| **Duration (years), mean (SD)** | 15.2 (8.2) | 12.3 (5.2) | 15.3 (7.8) | 13.4 (6.6) | 0.1580 |
| **BMI (Kg/m2), mean (SD)** | 28.2(5.9) | 25.2 (4.6) | 28.4(5.0) | 26.7(4.9) | 0.0270 |
| **Gestational weight gain (Kg), mean (SD)** | 10.4 (8.2) | 11.5(3.2) | 10.5 (6.7) | 10.6 (6.7) | 0.9348 |
| **First HBA1c (%), mean (SD)** | 6.3 (0.5) | 6.6 (0.3) | 8.3 (0.9) | 8.6 (1.3) | <0.001 |
| **Last HBA1c (%), mean (SD)** | 5.8 (0.5) | 7.1 (0.6) | 6.0(0.4) | 7.6 (0.9) | <0.001 |
| **Placental weight (grams), median (IQR)** | 730 (610-823) | 775 (643-908) | 628 (569-690) | 650 (543-740) | 0.009 |
| **Total Daily dose of insulin (Units), mean (SD)** | 73.4 (33.4) | 61.6 (28.5) | 75.6(41) | 83.4(41) | 0.088 |
| **Pre-term labor, n (%)** | 37 (69.8%) | 15 (65.2%) | 33 (62.3%) | 73 (53.7%) | 0.192 |
| **C-section, n (%)** | 34 (64.1%) | 10 (43.5%) | 38 (71.7%) | 97 (71.3%) | 0.054 |
| **Large for gestational age, n (%)** | 18 (34.0%) | 12 (52.2%) | 7 (13.2%) | 41 (37.5%) | 0.002 |
| **Small for gestational age, n (%)** | 3 (5.7%) | 1 (4.3%) | 1 (1.9%) | 6 (4.4%) | 0.798 |
| **Macrosomia, n (%)** | 5 (9.6%) | 4 (18.2%) | 0% | 10 (7.4%) | 0.041 |
| **Neonatal ICU admission, n (%)** | 14(26.4%) | 5 (21.7%) | 18 (34.0%) | 46 (33.8%) | 0.543 |

**Mediation Analysis Supplementary file**

Supplementary Table 2 :- Observed Information Matrix (OIM) Neonatal Weight in grams

| Structural | Coefficient | Standard Error | Z | P>\|z\| | 95% CI |
| --- | --- | --- | --- | --- | --- |
| Log Placental weight (grams) | 1345.9 | 169.3 | 7.9 | <0.001 | 1014.1, 1677.7 |
| Frist A1C | -47.2 | 35.9 | -1.3 | 0.18 | -117.6, 23.2 |
| Last A1c | 166.9 | 43.9 | 3.80 | <0.001 | 80.9, 253.0 |
| Maternal Age (Years) | -3.5 | 9.6 | -0.36 | 0.715 | -22.3, 15.3 |
| Ethnicity | -156.4 | 74.6 | 2.09 | 0.036 | -303.0, -9.9) |
| BMI (Kg/m2) | 19.0 | 7.7 | 2.46 | 0.014 | 3.8, 34.2 |
| Duration of DM(Years) | 2.4 | 5.7 | 0.43 | 0.667 | -8.7, 13.6 |
| Gestational weight gain (Kg) | 1.7 | 6.1 | 0.28 | 0.779 | -10.2, 13.6 |
| Gestational age (Weeks) | 131.8 | 20.0 | 6.57 | <0.001 | 92.5, 171.1 |
| Cons | -11407.3 | 1312.9 | 8.69 | <0.001 | -13980.7, -8834.0 |

Supplementary Table 3 :- Observed Information Matrix (OIM) Log Placental Weight (grams)

| Structural | Coefficient | Standard Error | Z | P>\|z\| | 95% CI |
| --- | --- | --- | --- | --- | --- |
| Frist A1C | -0.06 | 0.02 | -3.67 | <0.001 | -0.10, -0.03 |
| Last A1c | 0.04 | 0.02 | 1.77 | 0.077 | -0.004, 0.082 |
| Maternal Age (Years) | -.0009 | 0.005 | -0.19 | 0.846 | -0.010, 0.008 |
| Ethnicity | -0.063 | 0.037 | -1.69 | 0.091 | -0.136, 0 .010 |
| BMI (Kg/m2) | -0.006 | 0.004 | 1.63 | 0.104 | -0.014, 0.001 |
| Duration of DM (Years) | 0.0007 | 0.003 | 0.27 | 0.787 | -0.005, 0.006 |
| Gestational weight gain (Kg) | 0.008 | 0.003 | 2.69 | 0.007 | 0.002, 0.014 |
| Gestational age (Weeks) | 0.034 | 0.010 | 3.45 | 0.001 | 0.014, 0.052 |
| Cons | 5.71 | 0.449 | 12.7 | <0.001 | 4.82, 6.58 |

Supplementary Table 4 Significance testing of indirect effect (unstandardized) of the first A1c on neonatal weight.

| Estimates | Delta | Sobel | Monte Carlo |
| --- | --- | --- | --- |
| Indirect effect | -85.4 | -85.4 | -85.4 |
| Std. Error | 25.6 | 25.6 | 25.5 |
| z-value | -3.330 | -3.330 | -3.352 |
| p-value | 0.001 | 0.001 | 0.001 |
| 95% (CI) | -135.7, -35.1 | -135.7, -35.1 | -137.5, -39.0 |

Supplementary Table 5:- **Baron and Kenny's approach to testing mediation**

| STEP 1 | log placental weight(grams): firsta1c (X -> M) with B=-0.063 and p=0.0001. |
| --- | --- |
| STEP 2 | Neonatal weight (grams): log placental weight (grams) (M -> Y) with B=1345.883 and p=0.0001. |
| STEP 3* | Neonatal weight (grams) : firsta1c (X -> Y) with B=-47.168 and p=0.189 |
| RIT** | (85.411 / 132.579) = 0.644 |
| RIH*** | (85.411 / 47.168) = 1.811 |

* As STEP 1, STEP 2, and the Sobel's test above are significant and STEP 3 is not significant; the mediation is complete!

** RIT ( Ratio of indirect effect / total effect)

***RIH (Ratio of Indirect effect/Direct effect)
